# Accelerated ageing is associated with increased COVID-19 severity and differences across ethnic groups may exist

**DOI:** 10.3389/fpubh.2022.1034227

**Published:** 2022-12-13

**Authors:** Joshua Reeves, Jaspal S. Kooner, Weihua Zhang

**Affiliations:** ^1^Department of Epidemiology and Biostatistics, Imperial College London, London, United Kingdom; ^2^The Medical School, University of Sheffield, Sheffield, United Kingdom; ^3^Department of Cardiology, Ealing Hospital, London North West University Healthcare NHS Trust, London, United Kingdom; ^4^National Heart and Lung Institute, Imperial College London, London, United Kingdom; ^5^Imperial College Healthcare NHS Trust, London, United Kingdom; ^6^MRC-PHE Centre for Environment and Health, Imperial College London, London, United Kingdom

**Keywords:** COVID-19, severity, ageing, accelerated ageing, ethnicities, UK Biobank (UKB)

## Abstract

**Background:**

While increased age is an established risk factor for COVID-19, there is great heterogeneity in outcomes within age groups. This is because chronological age does not reflect health, unlike biological age. We intend to investigate the association between accelerated ageing and COVID-19 outcomes through the lens of three measures, namely phenotypic age acceleration (PhenoAgeAccel), telomere length (Adjusted T/S Ratio) and facial ageing, and to examine whether there are differences across ethnic groups.

**Methods:**

Taking participants from the UK Biobank, we associated accelerated ageing with severe COVID-19 outcomes, defined as COVID-related hospitalisation or death. Separate logistic regressions models were created for age and the three accelerated ageing-related variables, adjusting for a variety of covariates in each model. Multivariable logistic regression models were also created within White, Black, Asian and Other ethnic groups to assess for potential differing associations. Forward likelihood ratio logistic regression models were created to evaluate importance of the variables and to assess for patterns of association across the total population and ethnic groups.

**Results:**

After adjusting for all covariates, the odds ratio (OR) and 95% confidence interval (95% CI) of COVID-19 severe outcomes for age was 1.080 (1.074–1.086). After further adjusting age for the accelerated ageing variables, the ORs were 1.029 (1.020–1.039) for PhenoAgeAccel and 0.847 (0.772–0.929) for Facial Ageing's “Younger Than You Are” while Adjusted T/S ratio and “Older Than You Are” were statistically insignificant. The OR for age remained similar across ethnic groups. Both PhenoAgeAccel and younger facial ages in the White population and PhenoAgeAccel in the Black population had ORs of 1.031 (1.021–1.042), 0.853 (0.774–0.939), and 1.049 (1.001–1.100), respectively. Both Adjusted T/S Ratio and older facial ages showed statistical insignificance in all ethnicities. In forward logistic regression, age and PhenoAgeAccel were the age-related variables selected most frequently in all models.

**Interpretation:**

Accelerated ageing is associated with increased COVID-19 severity. The mechanisms at work here are likely immunosenescence and inflamaging. This association indicates that anti-ageing treatment may improve COVID-19 outcome. The results within ethnic groups and that of telomere length were inconclusive, but point to a need for future, more focused research on the topic.

## Background

The current Coronavirus disease (COVID-19) pandemic began in Wuhan, China in December 2019 (1). Since then, as of the 25^th^ May 2022, there have been over 6 million confirmed deaths globally and just under 200 000 confirmed deaths in the UK (2, 3). During this period, several risk factors associated with greater disease severity have been established. One of which principally is increased age, which has been strongly associated with increased risk of severe complications of COVID-19 (4). For example, mortality has been shown to increase exponentially from year 50, with those aged 85 years and older having a mortality risk 100-fold larger than those aged 50 and under (4, 5).

This is likely due to ageing's association with a weakened immune system and its general contribution to an increased susceptibility to mortality (6, 7). However, within age groups, there is great heterogeneity in COVID-19 outcomes and as such, it seems that chronological age is not sufficient in explaining severity risk (8). This is because chronological age does not reflect biological health within individuals, which at advanced ages can be highly varied (7, 8). Biological age, however, does capture the genetic and epigenetic changes that occur over a lifetime and due to this, has shown a greater association with all-cause mortality (7, 8). Therefore, by accounting for accelerated ageing, biological age may also be a stronger predictor for COVID-19 outcome (7).

Interestingly, this seems to be ever more present when examining ethnicity. Studies have shown that specific ethnicity groups, such as African Americans, seem to experience increased accelerated ageing and as such, significantly higher biological ages (9). Therefore, it can be theorised that within these groups, the effects of ageing on COVID-19 outcomes could be exacerbated. This may explain why specific ethnic groups tend to do worse than others with the viral infection. However, the differing effects of accelerated ageing within ethnicities on COVID-19 outcomes has, to our knowledge, yet to be studied.

Several measures/markers for biological age have been identified and developed around capturing the effects of accelerated ageing on the body. Three of these have been chosen to be reviewed within this study, by examining their own associations with severe COVID-19 outcomes. These are phenotypic age, telomere length and facial ageing.

Firstly, phenotypic age (PhenoAge) is a biological age estimate developed and proposed in Kuo et al. (5). Calculated with chronological age and 9 biomarkers, studies have already proved an association between a higher PhenoAge and severe COVID-19 outcomes (5). However, this association has yet to be examined within ethnic groups.

Secondly, telomere length (TL) has been proposed as a biomarker for biological age due to its connection to cellular proliferative capacity and its association with age (10–12). Telomeres are repetitive sections of nucleoprotein structures found at the ends of chromosomes which ensure genome integrity (13). TL shortens after each cellular division and is a key determinant in cellular life (14). This is because after telomeres reach critical length, the cells enter into senescence and are unable to replicate (14). Again, while an association between shorter telomeres and severe COVID-19 outcomes has been shown, it has not been truly studied within ethnicities (12).

Lastly, facial ageing has been proposed as another form of measuring accelerated ageing. This comes after a study revealed a positive relationship between facial appearance and risk of age-related diseases, most notably cardiovascular disease (15). However, to our knowledge, there has been no research done into its relationship with COVID-19 outcomes.

This study then intends to investigate the association between accelerated ageing and COVID-19 outcomes through the lens of three measures and to examine whether there are differences across ethnic groups. It will take its data from the UK Biobank's (UKB) December 2021 update, which offers information on over 500 000 participants (16). This study intends to be the largest to date on the topic of COVID-19 and accelerated ageing and, to our knowledge, will be the first to look at this association across ethnicities.

## Method

### Participants

Our study will take its participants from the UKB, which is a large prospective cohort study that has collected and continues to collect in-depth medical information on over 500 000 participants (16, 17). UKB data has been collected through a variety of methods, such as questionnaires, physical measures, genomic assays, urinary and blood tests and longitudinal follow-ups on health outcomes (16).

In our analysis, we have only included participants from assessment centres within England and have excluded those who died before 16^th^ March 2020, asked for removal from the study, or lost to follow-up. Baseline biological information was taken at point-of-assessment during 2006–2010 and participant disease status was confirmed from the linked hospital records system Hospital Episode Statistics (HES) (16, 18). COVID-19 data (ICD-10 code “U07.1” and “U07.2”) on positive case numbers were taken from the combination of test results provided by Public Health England, hospital records provided by HES and primary care data from TPP and EMIS (19). This was due to some participants having a clinically diagnosis of COVID-19 but without a record of a positive test. COVID-19 hospitalisation records were taken from HES and mortality was confirmed from the death registry. COVID-19 data used will cover from the UKBs first COVID-19 test date (16^th^ March 2020) to the point of the UKBs December 2021 update.

### Variables

#### Age

“Age at 16/03/20” used in this study is the participants age as of 16^th^ March 2020 measured in years and has been calculated from adding the time difference between this date and assessment date to the participants age-at-assessment. It has been included in analysis models as a constant to compare the accelerated ageing variables to.

#### Telomere length

Adjusted Leukocyte TL (LTL) is the variable used in this study (field code 22191). Relative LTL was measured using a quantitative PCR method, expressed as a ratio of telomere repeat copy to single copy gene copy number, relative to a standard sample, and then adjusted for technical parameter influence (Adjusted T/S Ratio). Further details on the measurement method are provided (20). For analysis, outliers (exceeding mean+/-3SD) were removed.

#### Phenotypic age acceleration

Phenotypic age (PhenoAge) was calculated using the formula referenced, with more detail on how this calculation was developed provided (5, 21). It is made up of nine biomarkers and chronological age and has been directly contrasted against age-at-assessment to create PhenoAge Acceleration (PhenoAgeAccel). This is a value measured in years which represents how much biologically older/younger a participant is compared to their chronological age (21). Again, for analysis, PhenoAgeAccel outliers (exceeding mean+/-3SD) were discarded.

#### Facial ageing

Facial ageing has been measured by UKB on a questionnaire (field code 1757) answered by the participants (22). The question asked “Do people say that you look:” with five available answers of “Younger than you are,” “Older than you are,” “About your age,” “Do not know” and “Prefer not to answer.” For analysis, participants with responses “Do not know” and “Prefer not to answer” were removed and those with “About your age” were used as the reference category.

### Outcomes defined

The outcome examined was severe COVID-19 outcomes (ICD-10 code “U07.1” and “U07.2”), which was defined as any participant with a COVID-19 related hospitalisation or COVID-19 related mortality, including both primary and secondary diagnoses (19). Our inclusion criteria required a participant to have either a positive COVID-19 test or a clinical diagnosis of the illness. Within this population, the control group were those who did not have the severe outcome, labelled as “Mild COVID-19 Outcome,” and our cases were those who had the outcome, labelled “Severe COVID-19 Outcome.”

### Statistical analysis

All statistical analysis was performed in SPSS. Univariable logistic regression was performed to analyse each variable individually. Each variable was then added separately into three multivariable logistic regression models, with an increasing number of covariates added into each model. The second model (M2) adjusted for sex and ethnicity. Initial UKB data had split participants into 6 ethnic groups: White, Mixed, Asian, Black, Chinese and Other (23). However, groups Mixed, Chinese and Other were combined under the umbrella term “Other” as these groups made up < 2% of total participants each and as such, had too few cases for significant analysis. Chinese was not included within the Asian ethnic group as it represents a different subgroup of the Asian population, notably South Asian origin. The third model (M3) further adjusted for Body Mass Index (BMI), smoking status, alcohol drinking status and Townsend deprivation index. Finally, the fourth model (M4) took into account comorbidities, adjusting for the presence of coronary heart disease (ICD-10 codes I121-I123), type 2 diabetes (ICD-10 code E11), hypertension (ICD-10 codes I10-I15), obesity (ICD-10 code E66) and respiratory conditions Asthma or COPD (ICD-10 codes J41-J45) from linked HES data. These comorbidities were chosen due to their high prevalence in COVID-19 patients and their strong association with a higher risk of severe COVID-19 outcomes (24–27). A fifth multivariable logistic regression model (M5) was created for each accelerated ageing variable to further adjust for age.

Models 3 and 4 were then replicated within the individual ethnicities in order to examine the effect of the variables within each isolated group. Both models were created due to the large reduction in total participants (over 10% in most cases) as a result of including comorbidities. Age was adjusted for in both model types (3 and 4) for all of the accelerated ageing ethnicity models.

Forward likelihood ratio logistic regression was also performed to assess the important variables and covariates within our models. We created three different models to assess how the addition of age and covariates impacted the accelerated ageing variables. The first model (F1) included only the accelerated ageing variables, the second model (F2) included these plus Age and in the third model (F3), covariates were added. These models were then repeated within the ethnicities to assess for changes in importance and also to identify any repeating patterns between these subgroups and the total population.

### Ethics approval

The UKB has ethical approval from the North West Multi-Centre Research Ethics Committee (MREC) as a Research Tissue Bank (RTB) approval of the UK (REC reference 21/NW/0157). No separate ethical approval was required.

## Results

By December 2021, 27,909 participants had either a positive test or a clinical diagnosis of COVID-19. Among them, 4,016 of these went on to experience severe outcomes while 23 893 participants only experienced mild outcomes. Within those who experienced severe COVID-19 outcomes, 1,016 participants died (25.3%). Those who had severe COVID-19 tended to be both chronologically and phenotypically older, have shorter telomeres and also have older looking faces than those that had mild COVID-19. They were also more likely to be male, from a non-white background, live in a less affluent area and have a slightly larger BMI. While severe COVID-19 cases were more likely to have either been previous or current smokers, they were also more likely to have stopped or never drank alcohol before. Lastly, they were much more likely to have a pre-existing disease, particularly hypertension and type 2 diabetes, than those who had milder symptoms. A more detailed summary of the participants characteristics is shown in Table 1.

**Table 1:** Characteristics of participants by case status.

**Trait**		**Outcome**	
		**Mild COVID-19 (*N* = 23,893)**	**Severe COVID-19 (*N* = 4,016)**
Age at 16/03/20 in years		65 (8)	71 (8)
PhenoAgeAccel in years		−0.71 (4.58)	1.32 (5.23)
Adjusted T/S ratio		0.84 (0.13)	0.81 (0.12)
Facial ageing	About your age	5,140 (23.2)	967 (27.1)
	Younger than you are	16,405 (74.2)	2,478 (69.5)
	Older than you are	574 (2.6)	122 (3.4)
Sex	Female	13,019 (54.5)	1,657 (41.3)
	Male	10,874 (45.5)	2,359 (58.7)
Ethnic background	White	21,864 (91.9)	3,559 (89.4)
	Other	530 (2.2)	92 (2.3)
	Asian	847 (3.6)	160 (4.0)
	Black	545 (2.3)	168 (4.2)
Body mass index (BMI) in kg/m^2^		27.79 (4.8)	29.81 (5.7)
Smoking status	Never	13,227 (55.6)	1,729 (43.6)
	Previous	8,131 (34.2)	1,666 (42.0)
	Current	2,421 (10.2)	572 (14.4)
Alcohol drinker status	Never	1,146 (4.8)	307 (7.7)
	Previous	717 (3.0)	263 (6.6)
	Current	21,968 (92.2)	3,420 (85.7)
Townsend deprivation index		−1.82 (−3.45–1.03)	−0.89 (−3.04–2.42)
Comorbidities:			
Coronary heart disease	No	20,476 (96.9)	3,637 (90.7)
Disease	Yes	660 (3.1)	372 (9.3)
Type 2 diabetes	No	19,511 (92.3)	2,858 (71.3)
	Yes	1,625 (7.7)	1,151 (28.7)
Hypertension	No	15,061 (71.3)	1,357 (33.8)
	Yes	6,075 (28.7)	2,652 (66.2)
Obesity	No	19,520 (92.4)	3,030 (75.6)
	Yes	1,616 (7.6)	979 (24.4)
Respiratory	No	18,281 (86.5)	2,702 (67.4)
	Yes	2,855 (13.5)	1,307 (32.6)

Data shown are N (%) for categorical variables or Mean (SD) for continuous variables. As the distribution of the variable Townsend Deprivation index was skewed, the data shown here is Median (Interquartile Range). Respiratory includes asthma and COPD.

Before analysis, correlation scores between the 3 continuous variables (Age at 16/03/20, PhenoAgeAccel and Adjusted T/S ratio) were calculated. PhenoAgeAccel and Age have no correlation with a Pearson's correlation coefficient r and its 95% confidence interval (95% CI) = 0.000 (−0.003–0.004), *p* = 0.901; Adjusted T/S ratio and Age were moderately correlated with r (95% CI) = −0.194 (−0.197– −1.91), *p* < 0.001; PhenoAgeAccel and Adjusted T/S ratio were weakly correlated with r (95% CI) = −0.047 (95% CI: −0.050– −0.043), *p* ≤ 0.001.

### Accelerated ageing-related variables

One univariable and three multivariable logistic regression models were created for each variable to assess their own association with the likelihood of severe COVID-19 outcomes. A fourth multivariable model was created for the accelerated ageing variables to further adjust for age. Results on the four variables assessed are provided in Table 2.

**Table 2:** Multivariable logistic regression, with increasing number of covariates adjusted for, predicting the likelihood of severe COVID-19 outcomes, each variable analysed separately.

**Variable**	**Age**	**PhenoAgeAccel**	**Adjusted T/S ratio**	**Facial ageing** ^**†**^	
				**Younger than you are**	**Older than you are**
	**OR (95%CI)**	**OR (95%CI)**	**OR (95%CI)**	**OR (95%CI)**	**OR (95%CI)**
Model 1	1.104 (1.099–1.109)***	1.090 (1.082–1.098)***	0.156 (0.117–0.209)***	0.803 (0.741–0.870)***	1.130 (0.918–1.390)
Model 2	1.108 (1.103–1.113) ***	1.084 (1.076–1.093) ***	0.162 (0.121–0.218) ***	0.835 (0.769–0.906) ***	0.982 (0.796–1.210)
Model 3	1.110 (1.105–1.115)***	1.061 (1.052–1.070)***	0.192 (0.142–0.260)***	0.903 (0.830–0.983)*	0.836 (0.673–1.039)
Model 4	1.080 (1.074–1.086)***	1.029 (1.020–1.038)***	0.338 (0.245–0.467)**	0.916 (0.837–1.004)	0.784 (0.619–0.993)*
Model 5	-	1.029 (1.020–1.039)***	0.815 (0.585–1.137)	0.847 (0.772–0.929)***	1.005 (0.788–1.282)

Dependent Variable, Severe COVID-19 outcomes; OR, odds ratio; CI, confidence interval.

Model 1: univariable (each single variable only).

Model 2: variable adjusted for sex and ethnicity.

Model 3: variable adjusted for sex, ethnicity, smoking status, alcohol drinking status, body mass index, and Townsend deprivation index.

Model 4: variable adjusted for sex, ethnicity, smoking status, alcohol drinking status, and comorbidities (coronary heart disease, type 2 diabetes, hypertension, obesity, asthma, and chronic obstructive pulmonary disease).

Model 5: variable adjusted for sex, ethnicity, smoking status, alcohol drinking status, comorbidities (coronary heart disease, type 2 diabetes, hypertension, obesity, asthma, and chronic obstructive pulmonary disease), and age.

^†^Reference category is “About your age”.

^*^p < 0.05; ^**^p < 0.01; ^***^p < 0.001.

Multivariable logistic regression analysis was then performed within each ethnicity to examine the association between each variable and the likelihood of severe COVID-19 outcomes within individual ethnic groups. Information on the breakdown of the characteristics of the participants within each ethnicity is provided in Supplementary Table 1. The results of the ethnic group multivariable models for each of the four variables are provided in Tables 3, 4.

**Table 3:** Multivariable logistic regression, specific to each ethnicity, predicting the likelihood of severe COVID-19 outcomes, adjusting for covariates (not including comorbidities).

**Variable**	**Age**	**PhenoAgeAccel**	**Adjusted T/S ratio**	**Facial ageing** ^**†**^	
				**Younger than you are**	**Older than you are**
	**OR (95%CI)**	**OR (95%CI)**	**OR (95%CI)**	**OR (95%CI)**	**OR (95%CI)**
White	1.111 (1.105–1.117)***	1.055 (1.045–1.065)***	0.696 (0.496–0.976)*	0.814 (0.742–0.892)***	1.251 (0.979–1.598)
Asian	1.078 (1.053–1.102)***	1.024 (0.982–1.067)	1.806 (0.407–8.024)	0.747 (0.473–1.179)	0.628 (0.280–1.406)
Black	1.127 (1.098–1.156)***	1.069 (1.023–1.118)**	0.673 (0.134–3.393)	0.568 (0.274–1.179)	0.687 (0.150–3.135)
Other	1.116 (1.081–1.152)***	0.988 (0.932–1.048)	2.079 (0.214–20.164)	0.864 (0.430–1.735)	1.286 (0.332–4.983)

Dependent Variable, Severe COVID-19 outcomes; OR, odds ratio; CI, confidence interval.

All models adjusted for sex, smoking status, alcohol drinking status, body mass index, and Townsend deprivation index.

Accelerated ageing variable models were also adjusted for age.

^†^Reference category is “About your age”.

^*^p < 0.05; ^**^p < 0.01; ^***^p < 0.001.

**Table 4:** Multivariable logistic regression, specific to each ethnicity, predicting the likelihood of severe COVID-19 outcomes, adjusting for covariates.

**Variable**	**Age**	**PhenoAgeAccel**	**Adjusted T/S ratio**	**Facial ageing** ^**†**^	
				**Younger than you are**	**Older than you are**
	**OR (95%CI)**	**OR (95%CI)**	**OR (95%CI)**	**OR (95%CI)**	**OR (95%CI)**
White	1.082 (1.076–1.088)***	1.031 (1.021–1.042)***	0.770 (0.542–1.095)	0.853 (0.774–0.939)**	1.094 (0.842–1.422)
Asian	1.045 (1.018–1.072)***	0.978 (0.936–1.023)	2.074 (0.449–9.584)	0.767 (0.473–1.244)	0.486 (0.208–1.136)
Black	1.079 (1.048–1.112)***	1.049 (1.001–1.100)*	0.543 (0.093–3.166)	0.654 (0.296–1.445)	0.489 (0.071–3.374)
Other	1.074 (1.037–1.112)***	0.983 (0.924–1.046)	3.406 (0.314–36.946)	0.673 (0.318–1.421)	0.809 (0.184–3.567)

Dependent Variable, Severe COVID-19 outcomes; OR, odds ratio; CI, confidence interval.

All models adjusted for sex, smoking status, alcohol drinking status, Townsend deprivation index, and comorbidities (coronary heart disease, type 2 diabetes, hypertension, obesity, asthma, and chronic obstructive pulmonary disease).

Accelerated ageing variable models were also adjusted for age.

^†^Reference category is “About your age”.

^*^p < 0.05; ^**^p < 0.01; ^***^p < 0.001.

**Figure 1:**
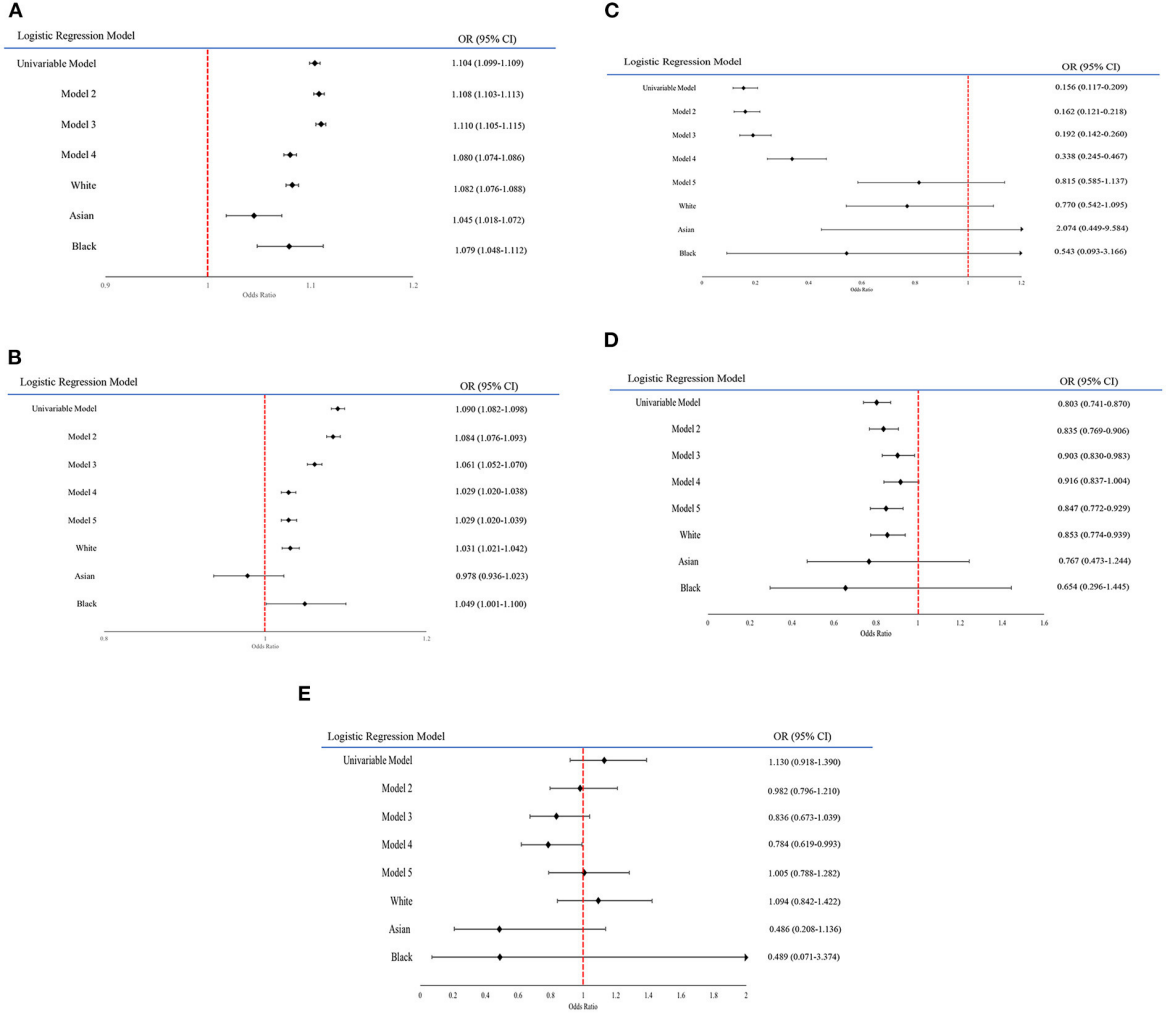
**(A–E)** Forest plot graphs displaying the changing odds ratios for each variable across the models. **(A)** A forest plot on the odds ratio of the variable Age within each model. **(B)** A forest plot on the odds ratio of the variable PhenoAgeAccel within each model. **(C)** A forest plot on the odds ratio of the variable Adjusted T/S within each model. **(D)** A forest plot on the odds ratio of the variable Facial Ageing “Younger than you are” within each model. **(E)** A forest plot on the odds ratio of the variable Facial Ageing “Older than you are” within each model.

Forest plots have been created to show the change in effect size across the models and ethnic groups (Figures 1A–E). However, ethnicity group “Other” is not included in the forest plots for each variable as some of the results were too large and would have disrupted the readability of the figures. In addition to this, the forest plots only include the ethnicity results from models adjusting for all covariates and age (results shown in Table 4).

#### Age

In univariable logistic regression (M1), increased age at the pandemic was associated with an increased risk of COVID-19 related hospitalisation and mortality, with an odds ratio (OR) and 95% CI = 1.104 (1.099–1.109, *p* < 0.001). After adjusting for sex and ethnicity in M2, the association slightly increased (OR = 1.108, 95% CI: 1.103–1.113, *p* < 0.001) and continued to increase after adjustment for smoking and alcohol status, BMI and Townsend deprivation in M3 (OR = 1.110, 95% CI: 1.105–1.115, *p* < 0.001). However, the association slightly attenuated in M4 after adjusting for comorbidities (OR = 1.080, 95% CI: 1.074–1.086, *p* < 0.001). These results were all statistically significant.

Across the ethnicities, increased age at the pandemic also showed consistently statistically significant association with an increased risk of COVID-19 severity. This was an association that was consistent even after adjusting for comorbidities, despite the effect size in all four ethnic groups attenuating. Within the White population, White model 1 (W1) had an OR = 1.111 (95% CI: 1.105–1.117, *p* < 0.001) while after adjusting for comorbidities in White model 2 (W2), the effect size slightly reduced (OR = 1.082, 95% CI:1.076–1.088, *p* < 0.001). Amongst Asian group, Age had an OR = 1.078 (95% CI: 1.053–1.102, *p* < 0.001) in Asian model 1 (A1), which slightly attenuated in Asian model 2 (A2) but remained significant (OR = 1.045, 95% CI: 1.018–1.072, *p* < 0.001). For the Black participants, Black model 1 (B1) had an OR = 1.127 (95% CI: 1.098–1.156, *p* < 0.001). However, the effect size slightly reduced in Black model 2 (B2) (OR = 1.079, 95% CI: 1.048–1.112, *p* < 0.001). In the Other group's first model (O1), Age had an OR = 1.116 (CI: 1.081–1.152, *p* < 0.001), which modestly decreased after adjusting for comorbidities in the second model (O2) (OR = 1.074, 95% CI: 1.037–1.112, *p* < 0.001).

#### PhenoAgeAccel

Univariable logistic regression showed that increased PhenoAgeAccel was significantly associated with increased likelihood of severe COVID-19 outcomes (OR = 1.090, 95% CI: 1.082–1.098, *p* < 0.001). This OR fell in M2 (OR = 1.084, 95% CI: 1.076–1.093, *p* < 0.001), continued to fall in M3 (OR = 1.061, 95% CI:1.052–1.070, *p* < 0.001) and finally in M4 (OR = 1.029, 95% CI: 1.020–1.038, *p* < 0.001), with all results being statistically significant. After adjusting for age in M5, however, the OR remained the same (OR = 1.029, 95% CI: 1.020–1.039, *p* < 0.001).

Ethnic groups White and Black both similarly found a positive relationship between increased PhenoAgeAccel and severe COVID-19 outcomes in their first models, with OR = 1.055 (95% CI: 1.045–1.065, *p* < 0.001) in W1 and OR = 1.069 (95% CI: 1.023–1.118, *p* = 0.003) in B1. After adjusting for comorbidities, the association in both ethnic groups remained consistent, demonstrating a positive association, with the OR in B2 = 1.049 (95% CI: 1.001–1.100, *p* = 0.046) and in W2 = 1.031 (95% CI: 1.021–1.042, *p* < 0.001). Both Asian and Other groups displayed statistically insignificant results in all four models.

#### Telomere length

Based on univariable logistic regression results, participants with longer LT (as in larger Adjusted T/S ratio) had a decreased risk of developing severe COVID-19 outcomes (OR = 0.156, 95% CI: 0.117–0.209, *p* < 0.001). This OR slightly increased after adjusting for sex and ethnicity in M2 (OR = 0.162, 95% CI: 0.162–0.209, *p* < 0.001), and again in M3 (OR = 0.192, 95% CI: 0.142–0.260, *p* < 0.001). It increased more considerably in M4 (OR = 0.338, 95% CI: 0.245–0.467, *p* < 0.001). However, after adjustment for age in M5, it became statistically insignificant.

Amongst the ethnicities, only W1 showed statistically significant associations. The model found a negative association with increased risk of developing severe COVID-19 outcomes, with OR = 0.179 (95% CI: 0.129–0.246, *p* < 0.001). All other models were statistically insignificant.

#### Facial ageing

Univariable logistic regression with facial ageing found that having a younger face had a statistically significant association with reduced risk of severe COVID-19 outcomes (OR = 0.803, 95% CI: 0.741–0.870, *p* < 0.001) while the OR for older faces was statistically insignificant. The OR for younger facial ages increased in the following two models, with the OR = 0.835 in M2 (95% CI: 0.769–0.906, *p* < 0.001) and OR = 0.903 in M3 (95% CI: 0.830–0.983, *p* = 0.019). While M4 showed a statistical insignificant result, the OR for younger facial ages showed statistical significance after adjusting for age in M5 (OR = 0.847, 95% CI: 0.772–0.929, *p* < 0.001). The opposite happened with older facial ages, where all models displayed statistical insignificance apart from M4, which found an association between older facial ages and a decreased risk of COVID-19 severity (OR = 0.784, 95% CI:0.619–0.993, *p* = 0.044).

Within ethnicities, there was only consistency found amongst results for younger facial ages within the white population. In these models, the ORs for younger facial ages in W1 (OR = 0.915, 95% CI: 0.838–1.000, *p* = 0.049) and W2 (OR = 0.853, 95% CI: 0.774–0.939, *p* = 0.001) showed negative associations with severe COVID-19 outcomes. The rest of the models were statistically insignificant.

### Forward likelihood ratio logistic regression

After performing forward likelihood ratio logistic regression, several patterns were identified. More details on the results of the models and the steps in which the variables were selected are shown in the Supplementary Tables 2–4.

#### Direction of association

The direction of association of the four variables remained almost completely consistent in all models. Age and PhenoAgeAccel had a positive association with increased COVID-19 severity in models that selected them. Similarly, if included, adjusted T/S ratio and younger facial ageing had consistent negative associations with increased likelihood of the outcome. However, older facial ageing was found to be negatively associated with severe COVID-19 outcomes in Black subgroup models, despite being found to be oppositely associated with the outcome in all other forward logistic regression models.

#### Position of selection in models

Again, the position of variable selection in the steps of the models were mostly consistent.

In F1, both in the entire population and in the White and Black subgroups, PhenoAgeAccel was selected first, Adjusted T/S ratio second and Facial Ageing third. However, in the Asian population, only PhenoAgeAccel was selected by the model and in the Other group, none were.

In F2, Age was selected first by every model and was the only variable selected in the Other group model. Although rejected by the Other subgroup model, PhenoAgeAccel was always the second variable selected in the other F2 models. The addition of Age into the models lead to Facial Ageing being selected before Adjusted T/S ratio in the White subgroup and total population. Both Facial Ageing and Adjusted T/S ratio were rejected in the Asian and Other group while Adjusted T/S ratio was also rejected from the Black F2 model.

In F3, Age was included in all models and was selected either first or second in all except the Asian group. PhenoAgeAccel was selected in all models except the Asian and Other F3 models and was the earliest selected out of all accelerated ageing variables. While Adjusted T/S ratio was rejected by all F3 models, Facial Ageing was included in the last step of the White and Black sub group models and in the final two steps of the total population model. Out of the covariates, aside from Coronary Heart Disease, Comorbidities were disproportionally selected earliest within all models. Hypertension was particularly selected highly, being selected first in all models except from the White and Asian groups, where it was selected second.

## Discussion

Overall, accelerated ageing has been proved to be associated with severe COVID-19 outcomes through the analysis of increased age and three ageing-related variables.

Firstly, increasing age has shown a positive association with the main outcome; an association consistent amongst all ethnicities. This alongside its strong appearance within the forward logistic regression models suggests it is a robust predictor for COVID-19 severity. This finding has supported the validity of our results as elder age is a known risk factor for COVID-19 and has been shown in several studies to be causally linked with an increased risk of COVID-related hospitalisation and mortality (28, 29).

Older biological age compared to chronological age (PhenoAgeAccel) has also shown a similar positive association. This link may potentially be causal as it remained positive and statistically significant even after adjusting for age and other covariates. In fact, PhenoAgeAccel has previously been shown to be linked to COVID-19 severity and may potentially be used in future predictive performance models for the viral illness, helping to identify severity risk in similar age groups (5). However, results look to be inconsistent amongst different ethnic groups. While it seems to be positively associated amongst White participants, the insignificant results within the main ethnic models and inconsistent selection within the forward logistic regression models suggest that PhenoAgeAccel as a variable isn't perfectly predictive for COVID-19 outcomes within all ethnicities. This as an area has not been explored and, as such, could be interesting for future research.

Individuals with shorter telomeres have not been proved in our study to have increased risk of severe COVID-19 outcomes. While this association was consistent through the four logistic regression models, it became insignificant after adjusting for age. There was also a large change in the effect size after adjusting for comorbidities in M4 and it was consistently rejected by the forward regression models after the addition of age and covariates into the model. This likely explains a lot of the association between TL and COVID-19 severity shown within these models and is possibly as a result of the established link between telomeres and these covariates. Firstly, age has been shown to negatively affect TL, with older age linked to shorter telomeres (12). Secondly, smoking has also been shown to negatively affect TL and likewise, so have the comorbidities; type 2 diabetes and obesity (30–32). Thirdly, research has also shown a link between short telomeres and hypertension, chronic obstructive pulmonary disorder and coronary heart disease–the other comorbidities included within M4 (33–35). Therefore, our results are unable to prove an independent association between long TL and COVID-19 severity as it seems the relationship is explained by covariates, despite previous findings from current research suggesting otherwise. Further research on any association within individual ethnic groups is also clearly needed. Studies have shown that with increasing age, telomeres shorten quicker in Black and Hispanic individuals compared to White individuals (36–38). While there are many influencing factors, this may contribute to why these ethnic groups tend to do worse with COVID-19. The statistical insignificance of our results however meant that we also were unable to prove this theory and therefore, this may benefit from a more directed investigation.

Finally, our results show for the first time a potential link between facial ageing and COVID-19 outcomes. While the results do not show that an ageing face is a risk factor of viral severity, they might suggest that a younger facial age is protective against the illness. Despite the insignificant result within M4, it returned to being statistically significant after adjusting for age and, like the other models, showed a negative association with the main outcome. This was a similar association also found in forward regression models. However, while it is unlikely that facial age directly impacts COVID-19 severity, excessive facial ageing is a sign of accelerated ageing and may point to internal mechanisms at work (39, 40). Within the ethnic groups, our study found results that suggest that potentially younger facial ages might be protective for the White population. However, within the Black and Asian group, there was a lack of significant results to form any conclusions on. While the results in W1 and W2 are consistent with the findings in M1, M2, M3 and M5, the findings within the other ethnic subgroups are inconclusive. However, this may be explained by Alexis et al. (41) which studied the differences in self-assessment of facial age by women in different ethnic groups (41). Within this study, it found that White women tended to report increased facial ageing earlier than both Asian and Black women by up to 20 years (41). Reasons for this varied between the effect of ultraviolet (UV) light on different skin pigmentations and differing usage of facial products (41). For example, Asian women tended to use more facial products like SPF sunscreen, while darker skin pigmentation reduced UV radiation penetration by approximately 50% compared to lighter skin (41). This study demonstrates how accelerated ageing may affect a individuals facial age differently due to their ethnicity, which might point to why there is an inconsistency and insignificancy within our results.

The mechanisms that likely underline the association between accelerated ageing and COVID-19 severity are multifaceted but are largely characterised by an increase in inflamaging and immunosenescence (5, 42, 43). Firstly, inflamaging is a process by where the body's proinflammatory status increases as a result of ageing (42). Accelerated ageing therefore leads to worsened inflamaging and a higher proinflammatory state (5). This has been shown to exacerbate COVID-19 symptoms by increasing the likelihood of severe inflammation, which could result in damaged tissue and a cytokine storm–a major cause of mortality in COVID-19 infections (5, 42–44). Secondly, immunosenescence is another process linked with accelerating ageing and short telomeres. Individuals with short telomeres have a reduced T cell proliferation ability and a higher proportion of senescent T cells (12, 43). This issue is then exacerbated by COVID-19, which is known to cause T cell lymphopenia (43). The individual's adaptive immunity is then severely weakened and unable to form the SARS-CoV-2 antigen-specific memory T cell which is crucial for viral clearance (43). Furthermore, the poor adaptive immune response leaves the innate response unmoderated and, can in turn, lead to hyperinflammation and again, a cytokine storm (43).

The association found in our study between accelerated ageing and severe COVID-19 outcomes is a consistent finding amongst similar papers and is in fact an association that is repeated in a plethora of viral illnesses and age-related diseases (5, 45). As such, it is clearly an area that could benefit from therapeutic interventions. It is possible that an intervention that works to slow or reverse the ageing process by targeting previously mentioned mechanisms may provide us with a new avenue to reduce the risk of viral infections, such as COVID-19. Therapeutic drugs such as rapamycin, which has shown to increase autophagy, and metformin, which improves insulin sensitivity, are all drugs with key anti-ageing properties that could be utilised in early prevention of COVID-19 (46, 47). Another type of drug that has been considered are senolytics, which work by chemically removing senescent cells and in turn, reducing inflammation (46, 47). Hydroxychloroquine and azithromycin are two drugs that work together like senolytics that are currently under clinical trials for the use in treatment of COVID-19 (47). Telomerase activation-based therapies, which maintain telomere length, is another opportunity that could be used to reduce the risk of COVID-related hospitalisation and mortality (7). Furthermore, some natural compounds such as polyphenols have proven anti-aging effects, which may be used as alternative therapeutic agents in combating age-related disease (48). Overall, our study corroborates previous research on the relationship between accelerated ageing and COVID-19 outcomes and adds to the clear indication for anti-ageing therapy for the viral infection.

Our study has several limitations. Firstly, the UKB sample is highly selective and has a healthier population than the UK public. For example, the cohort study had a response rate of only 5.5% and has, compared with the general population, less self-reported health conditions (49). The high selective nature of the sample does leave our study potentially subject to collider bias. In addition, the UKB has a majority of White participants (94%) and a minority of other ethnic groups that is not representative of the UK population (23). Additionally, this huge discrepancy makes it challenging to fully assess risk amongst different ethnicities. It is likely that the lack of power due to small sample sizes for ethnic minorities has resulted in non-significant results for these ethnic groups. Another limitation is that not all potential covariates were adjusted for, importantly cancer and vaccination status (50). There are also limitations of the variables assessed. Firstly, the role of telomere length as an ageing biomarker has been somewhat questioned due to its poor predictive performance, with studies showing DNA methylation epigenetic clocks to be more accurate biological age predictors (8, 51, 52). Secondly, PhenoAges were calculated from baseline values taken 10–15 years ago and as such, may not be as accurate as a PhenoAge calculated from more recent data. Lastly, the level of facial ageing was personally decided by the participants, which may subject the results to response bias. This may be why only 2.7% of participants within this trial had elder facial ages and as such, could be why the results for this variable were consistently insignificant. Finally, there are also limitations of the statistical methods used, namely forward logistic regression. This type of analysis can over exaggerate odds ratios and lower *p-*values, which could lead to making inaccurate conclusions if used inappropriately (53). This is why the study has refrained from specifying particular statistics and has instead detailed the results from these models as generally as possible. It has also been used in conjunction with the main logistic regression models and not as a separate piece of analysis used to create individual deductions in order to reduce the impact of this limitation.

## Conclusion

Our study has shown that accelerated ageing is associated with increased likeliness of severe COVID-19 outcomes, even after adjusting for covariates. While within ethnicities there was inconclusive evidence, there are signs that accelerated ageing may affect each ethnic group differently. This particularly area of our study then deserves future investigation, with specific focus on the differing effects of facial ageing within ethnicities. Furthermore, this study adds to the current research on accelerated ageing, and justifies the need for future research on anti-ageing treatment for all viral infections, specifically COVID-19.

## Data availability statement

The data analyzed in this study is subject to the following licenses/restrictions: The UK Biobank data is available to any bona fide researchers upon approved applications. Requests to access these datasets should be directed to https://www.ukbiobank.ac.uk.

## Ethics statement

The studies involving human participants were reviewed and approved by the North West Multi-Centre Research Ethics Committee (MREC), as a Research Tissue Bank (RTB) approval (https://www.ukbiobank.ac.uk/learn-more-about-uk-biobank/about-us/ethics). The patients/participants provided their written informed consent to participate in this study.

## Author contributions

This research was conducted and written up by JR. WZ worked as a senior author by delivering the data for the study from the UK Biobank and providing guidance and editing throughout the process. WZ and JK acted as senior authors by creating and organising the concept for the study. All authors contributed to the study and approved to the submitted version.
