# Supplementary material for: Accelerated ageing is associated with increased COVID-19 severity and differences across ethnic groups may exist
Source: Front Public Health. 2022 Dec 13;10:1034227. doi: 10.3389/fpubh.2022.1034227 (PMC9792858; doi:10.3389/fpubh.2022.1034227)
Supplement: Supplementary file 1 [file Data_Sheet_1.docx]

**Supplementary Material**

| **Supplementary Table 1.** Characteristics of participants by case status and ethnicity. | | | | | | | | | | | | | | | | | | | | | | | | | |  |  |
| --- | --- | --- | --- | --- | --- | --- | --- | --- | --- | --- | --- | --- | --- | --- | --- | --- | --- | --- | --- | --- | --- | --- | --- | --- | --- | --- | --- |
| Traits | | White | | | | | | Other | | | | | | Asian | | | | | | Black | | | | | |  |  |
| Outcomes | | Mild COVID-19 | | | | | Severe COVID-19 | Mild COVID-19 | | | Severe COVID-19 | | | Mild COVID-19 | | | Severe COVID-19 | | | Mild COVID-19 | | | Severe COVID-19 | | |  |  |
| **Age at 16/03/20** in years | | 65 (8) | | | | | 72 (8) | 61 (7) | | | 67 (8) | | | 62 (7) | | | 66 (8) | | | 60 (7) | | | 67 (9) | | |  |  |
| **PhenoAgeAccel** in years | | -0.76 (4.52) | | | | | 1.29 (5.18) | -0.45 (5.22) | | | -0.26 (5.44) | | | -0.11 (5.20) | | | 1.43 (5.58) | | | 0.12 (5.31) | | | 2.52 (5.85) | | |  |  |
| **Adjusted T/S ratio** | | 0.84 (0.12) | | | | | 0.81 (0.12) | 0.88 (0.13) | | | 0.85 (0.11) | | | 0.84 (0.12) | | | 0.84 (0.14) | | | 0.91 (0.14) | | | 0.88 (0.13) | | |  |  |
| **Facial Ageing** | About your age | | 4855 (22.0) | | | 897 (25.1) | | | | 77 (0.3) | | 15 (0.4) | | | | 166 (0.8) | | | 40 (1.1) | | | 33 (0.1) | | | 18 (0.5) | |  |
|  | Younger than you are | | 14 919 (67.6) | | | 2181 (61.4) | | | | 405 (1.8) | | 63 (1.8) | | | | 549 (2.5) | | | 90 (2.5) | | | 487 (2.2) | | | 131 (3.7) | |  |
|  | Older than you are | | 492 (2.2) | | | 102 (2.9) | | | | 14 (0.1) | | 4 (0.1) | | | | 54 (0.2) | | | 11 (0.3) | | | 12 (0.1) | | | 4 (0.1) | |  |
| **Sex** | Female | | 11 921 (50.1) | | | 1 452 (36.5) | | | | 316 (1.3) | | 44 (1.1) | | | | 397 (1.7) | | | 57 (1.4) | | | 324 (1.4) | | | 90 (2.3) | |  |
|  | Male | | 9 943 (41.8) | | | 2 107 (53.0) | | | | 214 (0.9) | | 48 (1.2) | | | | 450 (1.9) | | | 103 (2.6) | | | 221 (0.9) | | | 78 (2.0) | |  |
| **Body Mass Index (BMI)** in kg/m² | | 27.74 (4.79) | | | | | 29.71 (5.61) | 27.92 (5.03) | | | 29.47 (6.49) | | | 27.59 (4.75) | | | 29.46 (5.95) | | | 29.65 (5.64) | | | 32.07 (6.31) | | |  |  |
| **Smoking status** | Never | | 11 800 (49.8) | | | 1 450 (36.7) | | | | 320 (1.3) | | 43 (1.1) | | | | 689 (2.9) | | | 113 (2.9) | | | 383 (1.6) | | | 109 (2.8) | |  |
|  | Previous | | 7 783 (32.8) | | | 1 567 (39.7) | | | | 142 (0.6) | | 31 (0.8) | | | | 81 (0.3) | | | 22 (0.6) | | | 101 (0.4) | | | 40 (1.0) | |  |
|  | Current | | 2 220 (9.4) | | | 515 (13.1) | | | | 67 (0.3) | | 17 (0.4) | | | | 72 (0.3) | | | 22 (0.6) | | | 57 (0.2) | | | 17 (0.4) | |  |
| **Alcohol drinker status** | Never | | 533 (2.2) | | | 168 (4.2) | | | | 97 (0.4) | | 24 (0.6) | | | | 402 (1.7) | | | 85 (2.1) | | | 104 (0.4) | | | 30 (0.8) | |  |
|  | Previous | | 625 (2.6) | | | 240 (4.2) | | | | 29 (0.1) | | 6 (0.2) | | | | 38 (0.2) | | | 9 (0.2) | | | 23 (0.1) | | | 7 (0.2) | |  |
|  | Current | | 20 697 (87.1) | | | 3145 (79.2) | | | | 401 (1.7) | | 62 (1.6) | | | | 404 (1.7) | | | 68 (1.7) | | | 417 (1.8) | | | 129 (3.2) | |  |
| **Townsend deprivation index at recruitment** | | | | -2.02  (4.19) | -1.25  (5.14) | | | | 0.84 (5.81) | | | | 2.45 (5.54) | | 0.70 (4.57) | | | 1.58 (3.99) | | | 2.44 (5.66) | | | 3.65 (5.05) | | | |
| **Comorbidities:** | | | | | | | | | | | | | | | | | | | | | | | | | | | |
| **Coronary Heart Disease** | No | | 18 751 (89.1) | | | 3 223 (81.1) | | | | 454 (2.2) | | 85 (2.1) | | | | 705 (3.4) | | | 140 (3.5) | | | 477 (2.3) | | | 157 (4.0) | |  |
|  | Yes | | 606 (2.9) | | | 330 (8.3) | | | | 9 (<0.1) | | 7 (0.2) | | | | 33 (0.2) | | | 20 (0.5) | | | 8 (<0.1) | | | 10 (0.3) | |  |
| **Type 2 Diabetes** | No | | 18 050 (85.8) | | | 2 619 (65.9) | | | | 402 (1.9) | | 62 (1.6) | | | | 563 (2.7) | | | 71 (1.8) | | | 420 (2.0) | | | 87 (2.2) | |  |
|  | Yes | | 1 307 (6.2) | | | 934 (23.5) | | | | 61 (0.3) | | 30 (0.8) | | | | 175 (0.8) | | | 89 (2.2) | | | 65 (0.3) | | | 80 (2.0) | |  |
| **Hypertension** | No | | 13 916 (66.1) | | | 1 231 (31.0) | | | | 331 (1.6) | | 28 (0.7) | | | | 446 (2.1) | | | 49 (1.2) | | | 312 (1.5) | | | 36 (0.9) | |  |
|  | Yes | | 5 441 (25.9) | | | 2 322 (58.5) | | | | 132 (0.6) | | 64 (1.6) | | | | 292 (1.4) | | | 111 (2.8) | | | 173 (0.8) | | | 131 (3.3) | |  |
| **Obesity** | No | | 17 884 (85.0) | | | 2 701 (68.0) | | | | 426 (2.0) | | 69 (1.7) | | | | 688 (3.3) | | | 123 (3.1) | | | 438 (2.1) | | | 114 (2.9) | |  |
|  | Yes | | 1 473 (7.0) | | | 852 (21.5) | | | | 37 (0.2) | | 23 (0.6) | | | | 50 (0.2) | | | 37 (0.9) | | | 47 (0.2) | | | 53 (1.3) | |  |
| **Respiratory** | No | | 16 752 (79.6) | | | 2 367 (59.6) | | | | 392 (1.9) | | 72 (1.8) | | | | 642 (3.1) | | | 116 (2.9) | | | 414 (2.0) | | | 122 (3.1) | |  |
|  | Yes | | 2 605 (12.4) | | | 1 186 (29.9) | | | | 71 (0.3) | | 20 (0.5) | | | | 96 (0.5) | | | 44 (1.1) | | | 71 (0.3) | | | 45 (1.1) | |  |
| Data shown are N (%) for categorical variables or mean (SD) for continuous variables. As the distribution of the variable Townsend deprivation index was skewed, the data shown here is median (interquartile range). Respiratory includes conditions asthma and chronic obstructive pulmonary disease (COPD). | | | | | | | | | | | | | | | | | | | | | | | | | |  |  |

| Supplementary Table 2. A table showing the variable-related results from the first forward likelihood ratio logistic regression model which included the three accelerated ageing variables (Model F1). | | | | |
| --- | --- | --- | --- | --- |
|  | Variable | Step 1 | Step 2 | Step 3 |
| All | PhenoAgeAccel | 1.087 (1.078-1.096) *** | 1.086 (1.077-1.095) *** | 1.085 (1.076-1.094) *** |
|  | Adjusted T/S ratio |  | 0.174 (0.124-0.245) *** | 0.176 (0.125-0.248) *** |
|  |  |  |  |  |
|  | Facial Aging ^1^† |  |  | 0.838 (0.763-0.921) *** |
|  | Facial Aging ^2^† |  |  | 1.041 (0.815-1.329) |
| White | PhenoAgeAccel | 1.091 (1.081-1.100) *** | 1.089 (1.079-1.098) *** | 1.088 (1.078-1.097) *** |
|  | Adjusted T/S ratio |  | 0.159 (0.111-0.229) *** | 0.160 (0.111-0.230) *** |
|  |  |  |  |  |
|  | Facial Aging ^1^† |  |  | 0.841 (0.762-0.928) *** |
|  | Facial Aging ^2^† |  |  | 1.004 (0.769-1.309) |
| Asian | PhenoAgeAccel | 1.056 (1.015-1.099) ** |  |  |
|  | Adjusted T/S ratio |  |  |  |
|  |  |  |  |  |
|  | Facial Aging ^1^† |  |  |  |
|  | Facial Aging ^2^ |  |  |  |
| Black | PhenoAgeAccel | 1.088 (1.046-1.133) *** | 1.094 (1.050-1.140) *** | 1.097 (1.053-1.144) *** |
|  | Adjusted T/S ratio |  | 0.043 (0.008-0.247) *** | 0.044 (0.007-0.258) *** |
|  |  |  |  |  |
|  | Facial Aging ^1^† |  |  | 0.278 (0.133-0.579)  *** |
|  | Facial Aging ^2^† |  |  | 0.469 (0.115-1.922) |
| Other | PhenoAgeAccel |  |  |  |
|  | Adjusted T/S ratio |  |  |  |
|  |  |  |  |  |
|  | Facial Aging ^1^† |  |  |  |
|  | Facial Aging ^2^† |  |  |  |
| The table shows the variable-related results from the first forward likelihood ratio logistic regression model (F1) which included the accelerated ageing variables. It demonstrates the steps at which the variables were selected by the model, and their corresponding odds ratio (OR) and 95% confidence interval (CI).  Dependent variable: Severe COVID-19 outcomes.  † Reference category is “About your age”; 1: “Younger than you are”; 2: “Older than you are”.  *p<0.05; **p<0.01; ***p<0.001. | | | | |

| Supplementary Table 3. A table showing the variable-related results from the second forward likelihood ratio logistic regression model which included age and the accelerated ageing variables (Model F2). | | | | | |
| --- | --- | --- | --- | --- | --- |
|  | Variable | Step 1 | Step 2 | Step 3 | Step 4 |
| All | Age | 1.103 (1.097-1.109) *** | 1.102 (1.096-1.108) *** | 1.103 (1.097-1.109) *** | 1.102 (1.096-1.108) *** |
|  | PhenoAgeAccel |  | 1.083 (1.074-1.092) *** | 1.080 (1.071-1.090) *** | 1.080 (1.071-1.090) *** |
|  | Adjusted T/S ratio |  |  |  | 0.685 (0.479-0.979) * |
|  |  |  |  |  |  |
|  | Facial Aging ^1^† |  |  | 0.774 (0.701-0.853) *** | 0.776 (0.703-0.856)  *** |
|  | Facial Aging ^2^† |  |  | 1.439 (1.113-1.859) * | 1.432 (1.108-1.850) ** |
| White | Age | 1.108 (1.101-1.114) *** | 1.105 (1.099-1.112) *** | 1.107 (1.101-1.114) *** | 1.106 (1.099-1.112)  *** |
|  | PhenoAgeAccel |  | 1.084 (1.074-1.094)  *** | 1.081 (1.071-1.091) *** | 1.081 (1.071-1.091)  *** |
|  | Adjusted T/S ratio |  |  |  | 0.603 (0.412-0.882) ** |
|  |  |  |  |  |  |
|  | Facial Aging ^1^† |  |  | 0.765 (0.690-0.847)  *** | 0.767 (0.692-0.850)  *** |
|  | Facial Aging ^2^† |  |  | 1.429 (1.081-1.888) * | 1.415 (1.071-1.869) * |
| Asian | Age | 1.082 (1.053-1.112) *** | 1.081 (1.052-1.112)  *** |  |  |
|  | PhenoAgeAccel |  | 1.053 (1.011-1.097)  * |  |  |
|  | Adjusted T/S ratio |  |  |  |  |
|  |  |  |  |  |  |
|  | Facial Aging ^1^† |  |  |  |  |
|  | Facial Aging ^2^† |  |  |  |  |
| Black | Age | 1.126 (1.092-1.160) *** | 1.127 (1.093-1.163)  *** | 1.128 (1.093-1.165)  *** |  |
|  | PhenoAgeAccel |  | 1.092 (1.046-1.140)  *** | 1.098 (1.050-1.147)  *** |  |
|  | Adjusted T/S ratio |  |  |  |  |
|  |  |  |  |  |  |
|  | Facial Aging ^1^† |  |  | 0.249 (0.113-0.549) *** |  |
|  | Facial Aging ^2^† |  |  | 0.432 (0.088-2.114) |  |
| Other | Age | 1.124 (1.082-1.168) *** |  |  |  |
|  | PhenoAgeAccel |  |  |  |  |
|  | Adjusted T/S ratio |  |  |  |  |
|  |  |  |  |  |  |
|  | Facial Aging ^1^† |  |  |  |  |
|  | Facial Aging ^2^† |  |  |  |  |
| The table shows the variable-related results from the second forward likelihood ratio logistic regression model (F2) which included age and the accelerated ageing variables. It demonstrates the steps at which the variables were selected by the model, and their corresponding odds ratio (OR) and 95% confidence interval (CI).  Dependent variable: Severe COVID-19 outcomes.  † Reference category is “About your age”; 1: “Younger than you are”; 2: “Older than you are”.  *p<0.05; **p<0.01; ***p<0.001. | | | | | |

| Supplementary Table 4. A table showing the variable-related results from the third forward likelihood ratio logistic regression model which included age, accelerated ageing variables and all covariates (Model F3). | | | | | | | | | | | | | | |
| --- | --- | --- | --- | --- | --- | --- | --- | --- | --- | --- | --- | --- | --- | --- |
|  | Variable | 1 | 2 | 3 | 4 | 5 | 6 | 7 | 8 | 9 | 10 | 11 | 12 | 13 |
| All  (13 steps) | Age |  | 1.073 (1.066-1.079)  *** | 1.076 (1.070-1.083)  *** | 1.075 (1.068-1.081)  *** | 1.073 (1.067-1.080)  *** | 1.073 (1.066-1.079)  *** | 1.076 (1.069-1.083)  *** | 1.076 (1.070-1.083)  *** | 1.078 (1.071-1.084)  *** | 1.078 (1.071-1.084)  *** | 1.079 (1.072-1.086)  *** | 1.080 (1.073-1.087)  *** | 1.080 (1.073-1.086)  *** |
|  | PhenoAge Accel |  |  |  |  |  |  |  | 1.031 (1.021-1.041)  *** | 1.028 (1.018-1.038)  *** | 1.028 (1.018-1.038)  *** | 1.027 (1.017-1.037)  *** | 1.027 (1.017-1.037)  *** | 1.026 (1.016-1.036)  *** |
|  | Adjusted T/S ratio |  |  |  |  |  |  |  |  |  |  |  |  |  |
|  |  |  |  |  |  |  |  |  |  |  |  |  |  |  |
|  | Facial Aging ^1^† |  |  |  |  |  |  |  |  |  |  |  | 0.841 (0.756 -0.936)  ** | 0.841 (0.756-0.936)  ** |
|  | Facial Aging ^2^† |  |  |  |  |  |  |  |  |  |  |  | 1.075 (0.813-1.423) | 1.073 (0.811-1.420) |
| White  (11 steps) | Age | 1.102 (1.095-1.108)  *** | 1.077 (1.071-1.084)  *** | 1.081 (1.074-1.088)  *** | 1.079 (1.072-1.086)  *** | 1.078 (1.071-1.085)  *** | 1.077 (1.070-1.084)  *** | 1.079 (1.072-1.086)  *** | 1.079 (1.072-1.086)  *** | 1.081 (1.074-1.088)  *** | 1.080 (1.073-1.087)  *** | 1.081 (1.074-1.088)  *** |  |  |
|  | PhenoAge Accel |  |  |  |  |  |  |  | 1.033 (1.022-1.043) *** | 1.030 (1.019-1.040)  *** | 1.029 (1.018-1.039)  *** | 1.028 (1.018-1.039)  *** |  |  |
|  | Adjusted T/S ratio |  |  |  |  |  |  |  |  |  |  |  |  |  |
|  |  |  |  |  |  |  |  |  |  |  |  |  |  |  |
|  | Facial Aging ^1^† |  |  |  |  |  |  |  |  |  |  | 0.863 (0.773-0.964)  ** |  |  |
|  | Facial Aging ^2^† |  |  |  |  |  |  |  |  |  |  | 1.144 (0.848-1.543) |  |  |
| Asian  (6 steps) | Age |  |  |  |  | 1.039 (1.006-1.072)  * | 1.042 (1.009-1.077)  * |  |  |  |  |  |  |  |
|  | PhenoAge Accel |  |  |  |  |  |  |  |  |  |  |  |  |  |
|  | Adjusted T/S ratio |  |  |  |  |  |  |  |  |  |  |  |  |  |
|  |  |  |  |  |  |  |  |  |  |  |  |  |  |  |
|  | Facial Aging ^1^† |  |  |  |  |  |  |  |  |  |  |  |  |  |
|  | Facial Aging ^2^† |  |  |  |  |  |  |  |  |  |  |  |  |  |
| Black  (5 steps) | Age |  | 1.078 (1.041-1.116)  *** | 1.074 (1.036-1.113)  *** | 1.080 (1.041-1.120)  *** | 1.083 (1.044-1.124)  *** |  |  |  |  |  |  |  |  |
|  | PhenoAge Accel |  |  |  | 1.061 (1.011-1.114)  * | 1.064 (1.013-1.117)  * |  |  |  |  |  |  |  |  |
|  | Adjusted T/S ratio |  |  |  |  |  |  |  |  |  |  |  |  |  |
|  |  |  |  |  |  |  |  |  |  |  |  |  |  |  |
|  | Facial Aging ^1^† |  |  |  |  | 0.295 (0.123-0.709)  ** |  |  |  |  |  |  |  |  |
|  | Facial Aging ^2^† |  |  |  |  | 0.291 (0.043-1.988) |  |  |  |  |  |  |  |  |
| Other  (3 steps) | Age |  | 1.068 (1.023-1.115)  ** | 1.075 (1.029-1.123)  ** |  |  |  |  |  |  |  |  |  |  |
|  | PhenoAge Accel |  |  |  |  |  |  |  |  |  |  |  |  |  |
|  | Adjusted T/S ratio |  |  |  |  |  |  |  |  |  |  |  |  |  |
|  |  |  |  |  |  |  |  |  |  |  |  |  |  |  |
|  | Facial Aging ^1^† |  |  |  |  |  |  |  |  |  |  |  |  |  |
|  | Facial Aging ^2^† |  |  |  |  |  |  |  |  |  |  |  |  |  |
| The table shows the variable-related results from the third forward likelihood ratio logistic regression model (F3) which included age, accelerated ageing variables and all covariates (sex, smoking status, alcohol drinker status, Townsend deprivation index, and comorbidities). In “All” data analysis, ethnicity was also adjusted. Comorbidities included coronary heart disease, type 2 diabetes, hypertension, obesity, and respiratory (asthma or/and chronic obstructive pulmonary disease). It demonstrates the steps at which the variables were selected by the model, and their corresponding odds ratio (OR) and 95% confidence interval (CI).  Dependent variable: Severe COVID-19 outcomes.  † Reference category is “About your age”; 1: “Younger than you are”; 2: “Older than you are”.  *p<0.05; **p<0.01; ***p<0.001. | | | | | | | | | | | | | | |
